# Establishment of a prognostic ferroptosis- and immune-related long noncoding RNAs profile in kidney renal clear cell carcinoma

**DOI:** 10.3389/fgene.2022.915372

**Published:** 2022-08-30

**Authors:** Zhijun Han, Hao Wang, Yafei Liu, Xiao-Liang Xing

**Affiliations:** ^1^ Department of Urology, Department of Ultrasonography, Zhuzhou Hospital Affiliated to Xiangya school of Medicine, Central South University, Zhuzhou, China; ^2^ School of Public Health and Laboratory Medicine, Hunan University of Medicine, Huaihua, China; ^3^ Department of Urology, The First Affiliated Hospital, Hengyang Medical School, University of South China, Huaihua, China

**Keywords:** KIRC, ferroptosis, immune, lncRNAs, prognosis, diagnosis

## Abstract

**Background:** Ferroptosis and immunity are novel treatments that target several cancers, including kidney renal clear cell carcinoma (KIRC). Long noncoding RNAs (lncRNAs) are an important class of gene expression regulators that play fundamental roles in the regulation of ferroptosis and immunity. We aimed to identify ferroptosis- and immune-related lncRNAs as biomarkers in patients with KIRC.

**Methods:** Corresponding data for each patient with KIRC were obtained from The Cancer Genome Atlas (TCGA) database. Univariate and multivariate Cox regression analyses were used to identify candidate biomarkers followed by least absolute shrinkage and selection operator (LASSO) regression analyses, weighted gene coexpression network analysis (WGCANA), and gene set enrichment analysis (GSEA).

**Results:** Three ferroptosis- and immune-related differentially expressed lncRNAs (FI-DELs) (AC124854.1, LINC02609, and ZNF503-AS2) were markedly and independently correlated with the overall survival (OS) of patients with KIRC. The area under the curve (AUC) value of the prognostic model in the entire group using the three FI-DELs was > 0.70. The sensitivity and specificity of the diagnostic model using the three FI-DELs were 0.8586 and 0.9583, respectively.

**Conclusion:** The present study found that AC124854.1, LINC02609, and ZNF503-AS2 were considerably and independently correlated with the OS of patients with KIRC, suggesting that the three FI-DELs could be used as prognostic and diagnostic biomarkers for patients with KIRC.

## 1 Introduction

Kidney cancer is one of the most common malignant tumors originating in the kidneys. In 2020, approximately 180,000 deaths and 430,000 new cases of kidney cancer were reported globally (Sung et al., 2021). Almost 90% of kidney cancers are renal cell carcinoma (KIRC) (Singh, 2021). According to the morphology, renal cell carcinoma can be mainly divided into three subtypes: kidney renal clear cell carcinoma (KIRC), kidney renal papillary cell carcinoma, and kidney chromophobe (Owens, 2016). The most common subtype of kidney cancer is KIRC, and it accounts for approximately 80% of all primary malignant kidney tumors (Song et al., 2019; Zhou et al., 2020). Additionally, previous studies have demonstrated that 60% of patients with KIRC die within the first 2–3 years, and 30% of patients with KIRC are diagnosed with metastases (Mendoza-Alvarez et al., 2019). Moreover, numerous studies have demonstrated that the prognosis of patients with KIRC with metastases is poor (Chaffer and Weinberg, 2011; Forsea et al., 2012; Ljungberg et al., 2015). Currently, pathological tumors, nodes, and metastasis (TNM) are usually used as a biomarker to assess the risk of patients with KIRC (Wang et al., 2019a). Additionally, numerous studies have indicated that several molecular biomarkers, such as IL13RA2, SK1, and P4HB, can guide diagnosis, prognosis, and therapy (Salama et al., 2015; Shibasaki et al., 2015; Xie et al., 2020). However, the prognosis and diagnosis of KIRC remain poor. Therefore, it is necessary to obtain reliable biomarkers for the diagnosis and prognosis of patients with KIRC.

Ferroptosis is an iron-dependent form of cell death. Numerous studies have indicated that dysregulation of ferroptosis participates in the carcinogenesis and development of several cancers (Dixon et al., 2012; Nie et al., 2018). The regulation of ferroptosis contributes to anticancer therapy in various cancers, including drug-resistant cancers, such as non-small cell lung cancer and pancreatic cancer (Yamaguchi et al., 2013; Efferth, 2017). Immunotherapy targets specific cancer antigens and alerts the immune system to eradicate them through a concerted immune response (Johdi and Sukor, 2020). Immune cells and immune factors not only participate in antitumor immunity but also in the initiation and development of antitumor immunity (Berraondo et al., 2016; Chen et al., 2017). Cancer immunotherapy has been successfully used to treat many cancers (Im and Pavletic, 2017; Nixon et al., 2018). Interestingly, evidence indicates that there is a close link between ferroptosis and immunity. Ferroptosis cells can interact with immune cells such as NK cells and CD8+ T cells, among others, to release chemotaxis to regulate anticancer immunity (Wang et al., 2018a; Wang et al., 2019b; Stockwell and Jiang, 2019). Ferroptosis and immunity can regulate each other to participate in anticancer effects (Wang et al., 2019b; Stockwell and Jiang, 2019; Shi et al., 2021). The synergistic regulation of ferroptosis and immunity not only inhibits carcinogenesis but also stimulates immune responses (Li and Rong, 2020).

The lncRNAs are a group of transcriptional RNAs over 200 nucleotides in length that cannot encode proteins and are considered one of the most sensitive and specific cancer biomarkers (Fazal and Chang, 2016). Furthermore, they are involved in carcinogenesis and the development of various cancers (Wang et al., 2018b; Kim et al., 2018; Zhang et al., 2018; Slack and Chinnaiyan, 2019). Many lncRNAs can be used as prognostic biomarkers for several cancers, such as bladder cancer and lung cancer (Sun et al., 2020a; Zhang et al., 2021; Zhou et al., 2021). In this study, we evaluated FI-DELs as potential prognostic biomarkers using differential expression, Pearson correlation, and univariate/multivariate Cox regression analyses.

## 2 Materials and methods

### 2.1 Acquisition of corresponding data

The read counts data of KIRC (72 normal individuals and 530 patients with KIRC) and their corresponding clinical information (Table 1) were downloaded from an open database The Cancer Genome Atlas (TCGA) which do not need the approval of the local ethics committee. The read counts data of KIRC (45 normal individuals and 91 patients with KIRC) were downloaded from another open database International Cancer Genome Consortium (ICGC). DEseq2 in R (3.6.1) was used to screen the differentially expressed genes (DEGs) as the following criteria: baseMean ≥ 100, |log2FoldChange| ≥ 1.00, and *p.adj* < 0.05. The recognized lncRNAs, ferroptosis-related genes, and recognized immune-related genes were downloaded from the GENCODE database (https://portal.gdc.cancer.gov/), FerrDb database (http://www.zhounan.org/ferrdb), and ImmPort database (http://www.immport.org).

**TABLE 1:** Characteristics of KIRC patients.

Characteristic	Variable	Total (*n* = 530)	%
Age (years)	≤ 65	348	65.66
	> 65	182	34.34
Gender	Female	186	35.09
	Male	344	64.91
Stage	Stage I	265	50.00
	Stage II	57	10.75
	Stage III	123	23.21
	Stage IV	82	15.47
	Unknown	3	0.57
Tumor classification	T1	271	51.13
	T2	69	13.02
	T3	179	33.77
	T4	11	2.08
Lymph nodes	N0	239	45.09
	N1	16	3.02
	Unknown	275	51.89
Distant metastasis	M0	422	79.62
	M1	78	14.72
	Unknown	30	5.66
Survival status	Alive	357	67.36
	Death	173	32.64

Estimate in R (3.6.1) was used to evaluate the stromal, immune, and ESTIMATE scores and tumor purity. The evaluated infiltrating score of immune cells and immune factors was downloaded from Tumor IMmune Estimation Resource (TIMER) (https://cistrome.shinyapps.io/timer/).

### 2.2 Acquisition of ferroptosis- and immune-related signatures

After differentially expressed analyses, overlapping analyses were carried out for the differentially expressed genes (DEGs) with the recognized ferroptosis- and immune-related genes to screen the ferroptosis- and immune-related DEGs (FI-DEGs) and with the recognized lncRNAs to screen the DELs. Pearson correlation analyses were performed for the FI-DEGs and DELs to obtain the FI-DELs as the following criteria r ≥ 0.5 and *p* < 0.05.

### 2.3 Acquisition of prognostic biomarkers

The median value of each gene expression was used to regroup the patients with KIRC into low- and high-expression groups. Univariate Cox regression analyses followed by least absolute shrinkage and selection operator (LASSO) regression analyses were used to investigate the relationship of the FI-DELs with their overall survival (OS). Then, multivariate Cox regression analyses were performed to screen the suitable FI-DELs as biomarkers.

### 2.4 WGCNA and gene set enrichment analysis

The TIMER algorithm was utilized to estimate the stromal score, immune score, tumor purity, and ESTIMATE score. We used WGCNA that can convert coexpression correlation into connection weights, to identify coexpressed genes in stromal and immune cells (Langfelder and Horvath, 2008). Kyoto Encyclopedia of Genes and Genomes (KEGG) was used to assess the biological roles of the prognostic candidates by the “clusterProfiler” R package.

### 2.5 Construction of prognostic and diagnostic models

According to previous reports (Liu et al., 2020), the candidate biomarkers were used to construct a risk assessment model as follows: risk value = (0.831) × LINC02609 expression value + (−0.585) × ZNF503-AS2 expression value + (−0.530) × AC124854.1 expression value. A comprehensive index of ferroptosis and immune status (CIFI) was evaluated as follows: CIFI = (risk score—Min)/Max. The Youden index was used as the optimal cut-off value to regroup the patients with KIRC into low and high CIFI groups.

A diagnostic model was constructed as following after a stepwise logistic regression analyses: logit (P) = 0.747+ (0.212) × LINC02609 expression value + (−0.145) × ZNF503-AS2 expression value + (0.200) × AC124854.1 expression value (Liu et al., 2020). The Youden index was used as the optimal cut-off value to regroup the sample into a normal group and a KIRC group.

### 2.6 Statistical analyses

A repeated measure ANOVA followed by an unpaired two-tailed student’s t-test was used as indicated. All results were expressed as the mean ± SEM. Principal component analyses were used to reduce the dimensions and visualize the distribution of KIRC patients with different CIFI scores.

## 3 Results

### 3.1 Identification of ferroptosis- and immune-related differentially expressed lncRNAs as candidate biomarkers

Through differential expression analyses, we obtained 3,978 DEGs, including 2,573 upregulated and 1,405 downregulated DEGs (Supplementary Figure S1A). Of these, there were 531 FI-DEGs (405 upregulated FI-DEGs and 126 downregulated FI-DEGs) (Supplementary Figure S1B) and 361 DELs (278 upregulated DELs and 83 downregulated DGLs) (Supplementary Figure S1C). Pearson correlation analyses for the 531 FI-DEGs and 361 DELs found that there were 3,483 FI-DEGs–DELs pairs involving 362 FI-DEGs and 261 DELs. These 261 DELs were renamed FI-DELs.

**FIGURE 1:**
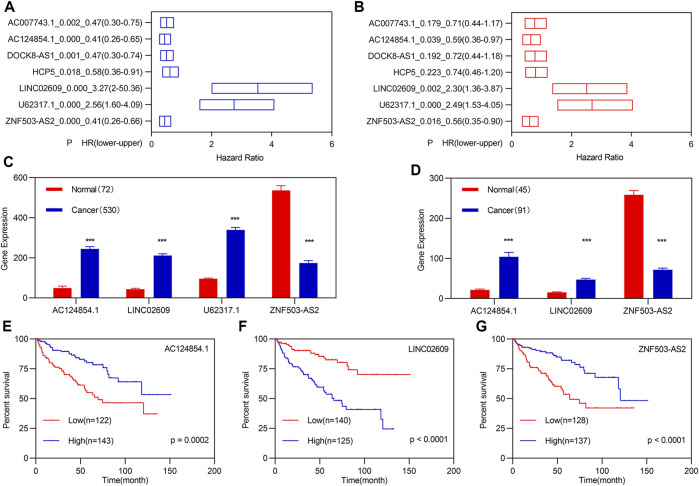
Identification of FI-DEGs as candidate biomarkers. **(A)** K-M analysis illustrated that seven FI-DELs were correlated with OS of patients with KIRC followed by LASSO analysis and WGCNA. **(B)** Multivariate Cox regression analysis illustrated that the four FI-DELs were correlated with the OS of patients with KIRC independently. **(C)** Expression of the four FI-DELs between normal and patients with KIRC in TCGA dataset. **(D)** Expression of the three FI-DELs between normal and patients with KIRC in the ICGC dataset. **(E–G)** K-M curve of the three FI-DELs in the training group. *p < 0.05, **p < 0.01, ***p < 0.001.

**FIGURE 2:**
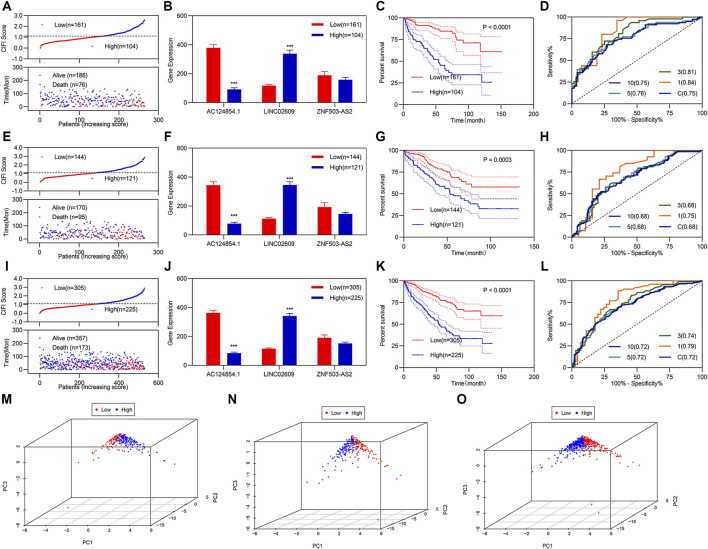
Development and validation of CIFI as a prognostic model. **(A–D)** and **(M)** Analysis of the prognostic model in the training group (*n* = 265). **(A)** For risk value (up) and survival status (down) for each patient. **(B)** Expression of the three candidate biomarkers. **(C)** K-M curve of KIRC patients with different CIFI values. **(D)** ROC curve of the risk assessment model. **(M)** Distribution of patients with different CIFI values. **(E–H)** and **(N)** Analysis of the prognostic model in the validation group (*n* = 265). **(E)** For risk value (up) and survival status (down) for each patient. **(F)** Expression of the three candidate biomarkers. **(G)** K–M curve of KIRC patients with different CIFI values. **(H)** ROC curve of the risk assessment model. **(N)** Distribution of patients with different CIFI values. i-l and o Analysis of the prognostic model in the entire group (*n* = 530). **(I)** For risk value (up) and survival status (down) for each patient. **(J)** Expression of the three candidate biomarkers. **(K) K–M** curve of KIRC patients with different CIFI values. **(L)** ROC curve of risk assessment model. **(O)** Distribution of patients with different CIFI values. *p < 0.05, **p < 0.01, ***p < 0.001.

All patients with KIRC (*n* = 530) were randomly divided into training (*n* = 265) and validation (*n* = 265) groups to verify and obtain suitable biomarkers. Univariate Cox regression analyses followed by LASSO analyses for the 261 FI-DELs in the training group were performed, and results indicated that 17 FI-DELs were correlated with the OS of patients with KIRC (Supplementary Figure S1D–F). Through ESTIMATE analyses in R software (3.6.1), we found that the stromal, immune, and ESTIMATE scores were considerably increased, while tumor purity was markedly decreased in patients with KIRC (Supplementary Figure S2A–D). In the TCGA-KIRC cohort dataset, we obtained 38 modules using WGCNA (Figures 2E,F). Of which, six modules and seven models were highly correlated with stromal and immune scores, respectively, which enriched 2,858 lncRNAs. By overlapping analyses with the 17 FI-DELs, we obtained seven FI-DELs (Figure 1A). Next, multivariate Cox regression analyses for the seven FI-DELs were performed, and the results indicated that four FI-DELs (AC124854.1, LINC02609, U62317.1, and ZNF503-AS2) remained independently correlated with the OS of patients with KIRC (Figure 1B). The expression of AC124854.1, LINC02609, and U62317.1 was significantly increased, whereas the expression of ZNF503-AS2 was markedly decreased in patients with KIRC (Figure 1C). To clarify the expression of those four candidate prognostic biomarkers in KIRC, we selected other independent samples for verification. The expressions of AC124854.1, LINC02609, and ZNF503-AS2 in the ICGC dataset were consistent with that in the TCGA dataset (Figure 1D). There was no expression of U62317.1. Therefore, only the candidates AC124854.1, LINC02609, and ZNF503-AS2 were selected for the subsequent analyses. Patients with a high expression of LINC02609 displayed worse OS, while patients with high expression of AC124854.1 and ZNF503-AS2 displayed better OS (Figures 1E–G).

### 3.2 Construction and validation of CIFI as a prognostic model

Based on previous studies, we constructed a risk assessment model using the three FI-DELs. The Youden index was used as the optimal cut-off value to regroup patients with KIRC (Supplementary Figure S3). The CIFI value and survival status of each patient with KIRC are shown in Figure 2A. In patients with KIRC having high CIFI values, the expression of LINC02609 was markedly increased, the expression of AC124854.1 was considerably decreased, and there was no substantial difference for ZNF503-AS2 (Figure 2B). Patients with KIRC and high CIFI values displayed a decreased OS (Figure 2C). Receiver operating characteristic (ROC) curve analyses indicated that the AUC value of this prognostic model was 0.75 (Figure 2D). Time-dependent ROC analyses indicated that the AUC values at 1, 3, 5, and 10 years were 0.84, 0.81, 0.76, and 0.75, respectively (Figure 2D). Principal component analyses (PCA) showed that patients with different CIFI values could be well distinguished using the three FI-DELs (Figure 2M).

Verification regarding the three FI-DELs as feasible biomarkers was performed by validation studies in the validation and entire groups; which indicated similar results for the validation and entire groups (Figures 2E–L). Particularly, the AUC values were 0.68 and 0.72 in both the validation group and the entire group respectively (Figures 2H,L). PCA showed that patients with different CIFI values could be well distinguished using the three FI-DELs in the validation and entire group (Figures 2N–O).

### 3.3 Independent prognostic model of the three ferroptosis- and immune-related differentially expressed lncRNAs

**FIGURE 3:**
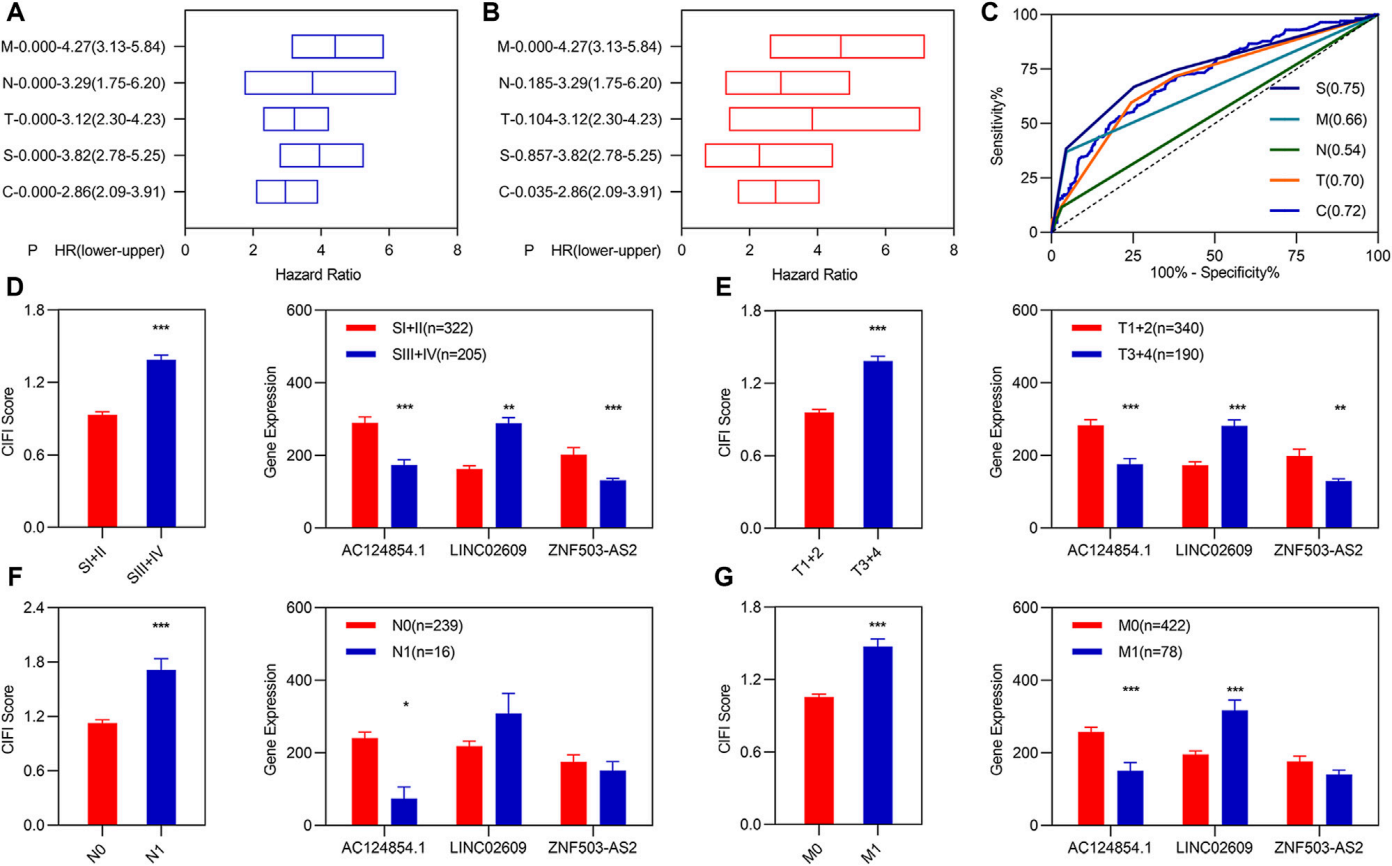
Independent prognostic values of the three FI-DELs. **(A)** K-M analysis illustrated different clinical characteristics, and CIFI values were correlated with OS of patients with KIRC. **(B)** Multivariate Cox regression analysis illustrated pathologic M, and CIFI values were correlated with the OS of patients with KIRC independently. **(C)** ROC curve of different clinical characteristics and CIFI value. **(D–G)** CIFI score and expression of the three FI-DELs within different clinical characteristics. *p < 0.05, **p < 0.01, ***p < 0.001.

Previous studies have demonstrated that TNM classification and stage are usually used as predictors to assess the risk in patients with KIRC. To determine whether the CIFI score was an independent prognostic factor for patients with KIRC, we performed univariate and multivariate Cox regression analyses among the clinical characteristics and CIFI scored in the entire group. Univariate Cox regression analyses showed that the CIFI score, pathologic TNM, and pathologic stage were significantly associated with the OS of KIRC (Figure 3A). Multivariate Cox regression analyses showed that the CIFI score and pathologic M were still significantly and independently associated with the OS of KIRC (Figure 3B). Subsequently, we plotted the ROC curve of the CIFI score and the different clinical characteristics and found that the AUC value of the CIFI score was higher than that of pathologic M (Figure 3C).

Correlation analyses showed that AC124854.1 and LINC02609 were significantly correlated with the CIFI values (Supplementary Figure S4). We carried out expression analyses to determine the relationship between the three FI-DEGs, CIFI scores, and clinical characteristics. The results are presented in Figures 3D–G. For example, the CIFI score was markedly associated with the pathologic TNM and stage (Figures 3D–G left). The expression of AC124854.1 was associated with the pathologic TNM, stage, and CIFI score (Figures 3D–G right).

### 3.4 Correlation analyses of CIFI with the immune cells and factors

**FIGURE 4:**
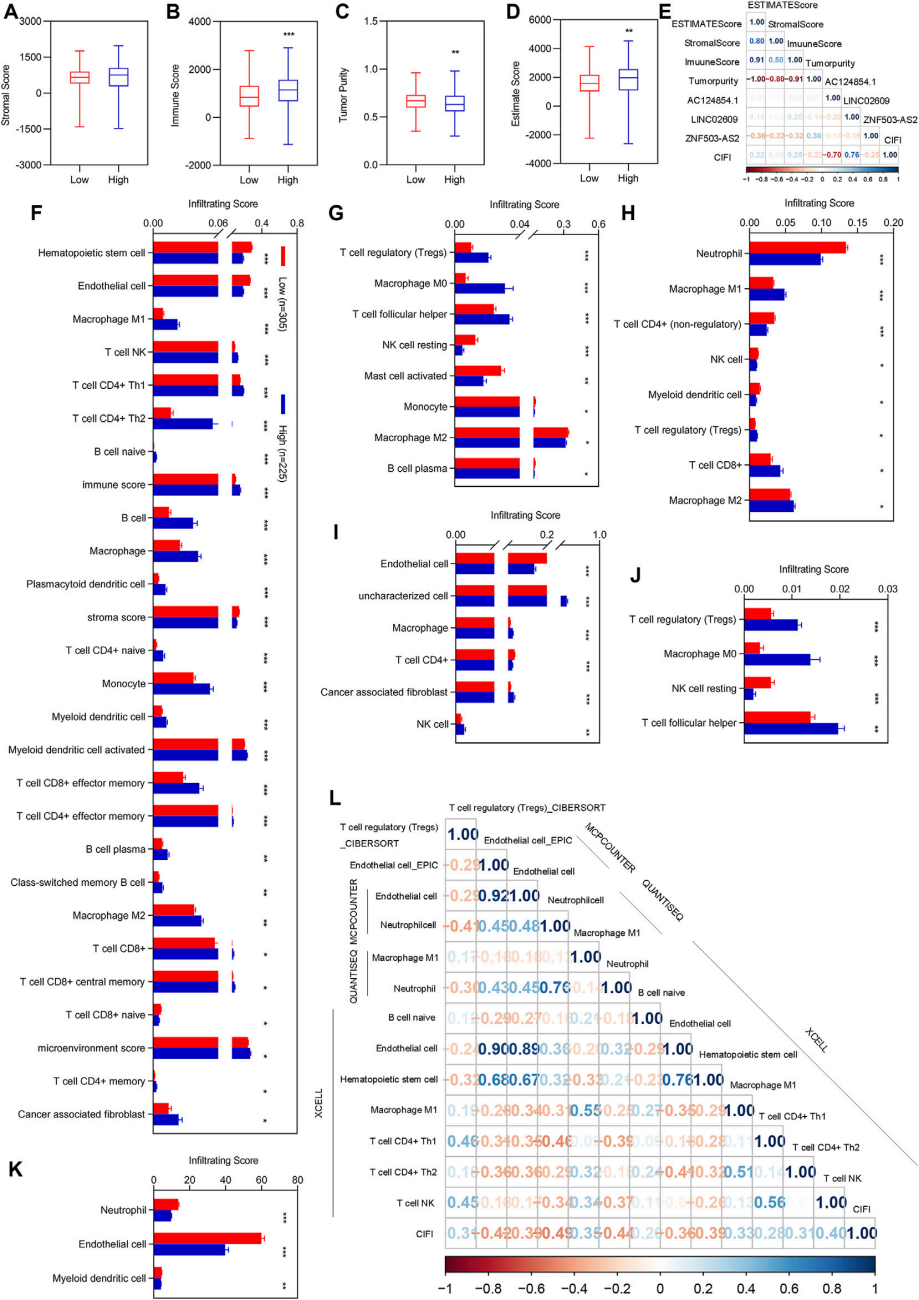
Correlation analysis of CIFI with the ESTIMATE score and immune infiltration. **(A–D)** Immune score between patients with KIRC with low CIFI value and patients with KIRC with high CIFI value. **(A)** Stromal score. **(B)** Immune score. **(C)** Tumor purity. **(D)** ESTIMATE score. **(E)** Correlation analysis of the three FI-DELs with the ESTIMATA Score. **(F–K)** Differentially expression analysis of the infiltrating score between patients with KIRC with low CIFI value and patients with KIRC with high CIFI value. **(F)** XCELL. **(G)** CIBERSORT. **(H)** quanTIseq. **(I)** EPIC. **(J)** CIBERSORT-ABS. **(K)** MCPCOUNTER. **(H)** Significantly associated immune cells and factors with CIFI. Correlation analysis of the prognostic model with the immune cells and factors. *p < 0.05, **p < 0.01, ***p < 0.001.

After regrouping, we re-evaluated the stromal score, immune score, tumor purity, and ESTIMATE score between different clusters. There was no significant difference in stromal scores between patients with high and low CIFI values (Figure 4A). The immune and ESTIMATE scores were significantly increased, while tumor purity was significantly decreased in patients with KIRC with high CIFI values (Figures 4B–D). We performed correlation analyses for the estimated score with the three candidate biomarkers and CIFI values for patients with KIRC with high and low CIFI values (Figure 4E).

To determine which immune cells and immune factors were correlated with CIFI, differential expression analyses were first performed. There were 88 immune cells and immune factors that were markedly different between normal individuals and patients with KIRC (Supplementary Table S1). Of these, 56 immune cells and factors were considerably different between patients with KIRC with high and low CIFI values (Figures 4F–K). To further determine the relationship among these 56 immune cells and factors with CIFI values, Pearson correlation analyses were performed and 13 out of 56 immune cells and factors were correlated with the CIFI (Figure 4l).

### 3.5 Construction of logit (P) as a diagnostic model and enrichment analyses of Kyoto Encyclopedia of Genes and Genomes

**FIGURE 5:**
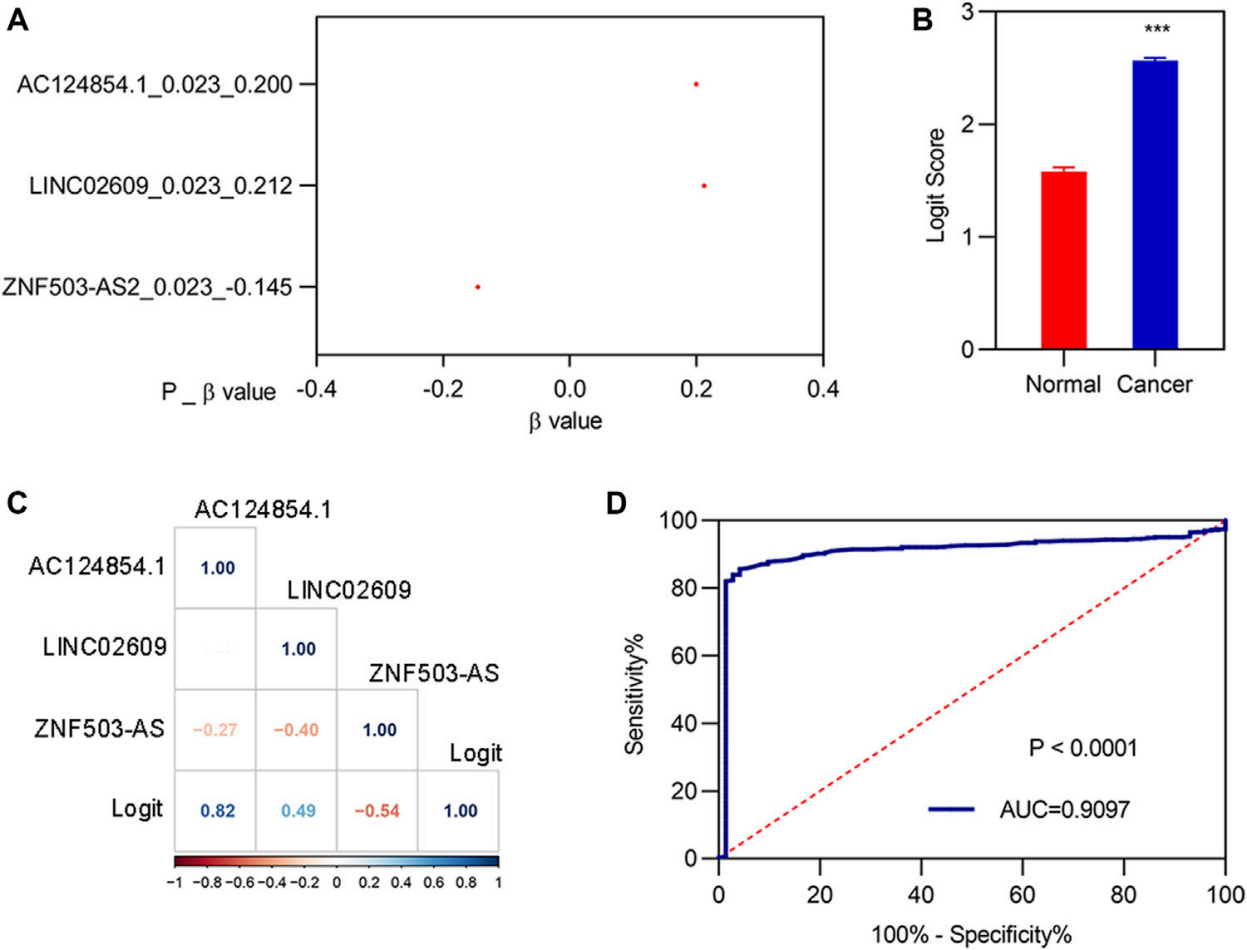
Construction of the diagnostic model. **(A)** β-value of the analysis of three FI-DELs by stepwise logistic regression. **(B)** Diagnosis values between normal and patients with KIRC. **(C)** Correlation analysis of the three FI-DELs with diagnosis values. **(D)** ROC curves of the diagnostic model. ***p < 0.001.

To determine whether the three biomarkers could be used for the diagnosis of patients with KIRC, a diagnostic model was constructed and assessed. Stepwise logistic regression for the three FI-DELs (Figure 5A) was performed. The diagnosis score was markedly increased in patients with KIRC (Figure 5B) and was markedly correlated with AC124854.1, LINC02609, and ZNF503-AS2 (Figure 5C). The sensitivity and specificity of the diagnosis were 0.8566 and 0.9583, respectively (Table 2). The AUC value of this diagnostic model was 0.9097 (Figure 5D).

**TABLE 2:** Sensitivity and specificity of the diagnostic model.

	Real KIRC	Real normal
Predicted KIRC	454	3
Predicted normal	76	69
Total	530	72
Correct	454	69
Sensitivity	0.8566	
Specificity		0.9583

**FIGURE 6:**
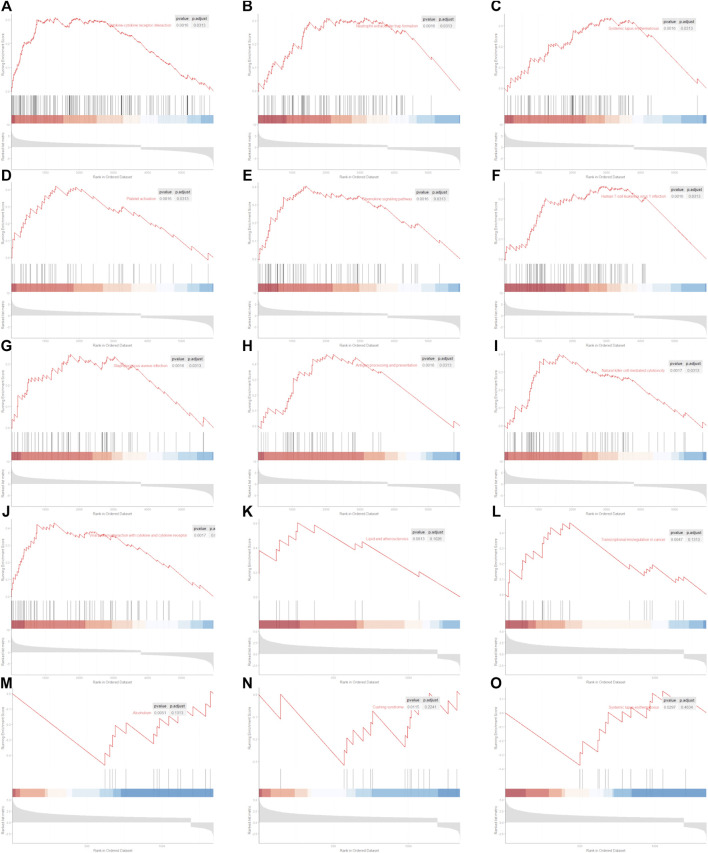
Significantly enriched KEGG pathway. **(A–J)** Top 10 significantly enriched signaling pathways between normal and KIRC. **(A)** Cytokine–cytokine receptor interaction. **(B)** Neutrophil extracellular trap formation. **(C)** Systemic lupus erythematosus. **(D)** Platelet activation. **(E)** Chemokine signaling pathway. **(F)** Human T-cell leukemia virus 1 infection. **(G)** *Staphylococcus aureus* infection. **(H)** Antigen processing and presentation. **(I)** Natural killer cell-mediated cytotoxicity. **(J)** Viral protein interaction with cytokine and cytokine receptor. **(K–O)** Five significantly enriched signaling pathways between patients with KIRC with low CIFI value and patients with KIRC with high CIFI value. **(K)** Lipid and atherosclerosis. **(L)** Transcriptional misregulation in cancer. **(M)** Alcoholism. **(N)** Cushing syndrome. **(O)** Systemic lupus erythematosus.

GSEA in R was used to compare the KEGG pathway between different clusters. We found that 60 signaling pathways were significantly enriched using the differentially expressed genes between normal and KIRC patients. The top 10 signaling pathways are shown in Figures 6A–J. We found that only five signaling pathways were significantly enriched using the differentially expressed genes between low CIFI and high CIFI patients (Figure 6K-6O). However, the signaling pathways they enriched were quite different.

## 4 Discussion

Kidney cancer is a heterogeneous disease. KIRC is one of the most common subtypes of kidney cancer and is characterized by high morbidity, mortality, and poor prognosis worldwide (Zhao et al., 2018; Mendoza-Alvarez et al., 2019). Due to the limited biomarkers in prediction, some patients with KIRC may be diagnosed with inaccurate grades, which could influence their OS (Wang et al., 2019a). Therefore, identifying new suitable biomarkers with high sensitivity and specificity is crucial for the prognosis and diagnosis of KIRC.

In the current study, gene expression data and clinical information of KIRC were obtained from TCGA. We obtained 261 FI-DELs through differential expression and correlation analyses. For the 261 FI-DELs, we performed univariate Cox regression analyses, multivariate Cox regression analyses, and WGCNA and found that three FI-DELs could be used as biomarkers for patients with KIRC. We constructed prognostic and diagnostic models using the three FI-DELs. Univariate and multivariate Cox regression analyses indicated that the prognostic model using the three FI-DELs was an independent prognostic factor. In addition, we constructed a diagnostic model using the three FI-DELs. The AUC value of the diagnostic model was 0.9097, indicating that this diagnostic model may be feasible. Previous studies have indicated that the three biomarkers identified in the present study were related to OS in other cancers. For example, Wu et al. (2020) found that ZNF503-AS2 could be used as an independent prognostic biomarker for rhabdoid tumors of the kidney by univariate and multivariate Cox analyses. Xing et al. (2021) found that LINC02609 was associated with the OS of patients with KIRC. Cao et al. (2021) found that the glycolysis-related lncRNA AC124854.1 was markedly correlated with the OS of renal cancer by univariate and multivariate Cox regression analyses. Cheng et al. (2019) also found that AC124854.1 could be used as a prognostic and diagnostic biomarker for KIRC. Our results are consistent with those of previous studies that reinforced the feasibility of our results.

Previous studies have found that surgery is the primary treatment for patients with KIRC because most patients are resistant to radiation and chemotherapy (Zhao et al., 2018; Evelonn et al., 2019; Yin et al., 2019). Discovering recently, ferroptosis and immunity are new therapeutic targets for cancer. Additionally, some prognostic markers based on ferroptosis and immunity also exist. For example, the AUC value of the risk assessment model constructed by Sun et al. using immune-related lncRNA signatures was 0.71 ^45^, and Xing et al. found that the AUC value of the risk assessment model constructed using the ferroptosis-related lncRNA signatures was 0.72 ^40^. We compared the present prognostic lncRNA features with published predictive models in patients with KIRC. Yin et al. (2018) found that nine lncRNAs (SLC25A5-AS1, COL18A1-AS1, WT1-AS, AC016773.1, LINC00460, LINC00313, HOTTIP, FGF14-AS1, and AC10502.1) could serve as independent biomarkers to predict survival in patients with KIRC. Sun et al. (2020b) found five immune-related lncRNA signatures (AC008105.3, LINC02084, AC243960.1, AC093278.2, and AC108449.2) with the ability to predict the prognosis of patients with KIRC. Liu et al. (2018) found that 19 lncRNAs (LOC606724, SCART1, SNORA8, LOC728024, HAVCR1P1, FCGR1CP, LINC00240, LINC00894, GK3P, SNHG3, KIAA0125, URB1-AS1, ZNF542P, TINCR, LINC00926, PDXDC2P, COL18A1-AS1, LINC00202-1, and LINC00937) that are potential biomarkers for the prognosis of KIRC. The AUC values of the three models were 0.684, 0.709, and 0.723, respectively, which were slightly lower than those of the present study (Liu et al., 2018; Yin et al., 2018; Sun et al., 2020b). Additionally, Zhang et al. (2019) found that 11 lncRNA signatures (AC245100.1, AP002761.1, LINC00488, AC017033.1, LINC-PINT, COL5A1-AS1, AC026471.4, AL009181.1, AL078590.3, LINC00524, and HOTTIP) could be potential biomarkers for KIRC. Yu et al. (2021) found that five prognostic-associated m6A-related lncRNAs (AC012170.2, AL157394.1, AP006621.2, AC025580.3, and AC124312.5) can be used as prognostic biomarkers for KIRC. The AUC values of these models were 0.781, and 0.809 respectively, which were slightly higher than those in the present study (Zhang et al., 2019; Yu et al., 2021). Comparatively, these two models used more prognostic biomarkers than those used in this study.

Although previous studies and our results indicate that the three FI-DELs may be used as prognostic and diagnostic biomarkers for patients with KIRC, the lack of cross-validation of other independent data and prospective clinical validation is an important shortcoming of our study. In addition, *in vitro* cell experiments and *in vivo* animal experiments are necessary to understand the functions of the three FI-DELs as biomarkers.

## 5 Conclusion

Through a series of bioinformatics analyses, we found that three FI-DELs (AC124854.1, LINC02609, and ZNF503-AS2) were independently significantly correlated with the OS of patients with KIRC. The prognostic and diagnostic models suggested that three FI-DELs could be used as prognostic and diagnostic biomarkers in patients with KIRC.

## Data Availability

Publicly available datasets were analyzed in this study. These data can be found at: https://portal.gdc.cancer.gov/.
