# Supplementary material for: Establishment of a prognostic ferroptosis- and immune-related long noncoding RNAs profile in kidney renal clear cell carcinoma
Source: Front Genet. 2022 Aug 30;13:915372. doi: 10.3389/fgene.2022.915372 (PMC9468637; doi:10.3389/fgene.2022.915372)
Supplement: Supplementary file 1 [file DataSheet1.pdf]

Establishment of a prognostic ferroptosis- and immune-related long noncoding RNAs profile in kidney renal clear cell carcinoma

Zhijun Han[1#], Hao Wang[1,3#], Yafei Liu1, Xiao-Liang Xing[1,2*]

[1]Department of Urology, Department of Ultrasonography, Zhuzhou Hospital Affiliated to Xiangya school of Medicine, Central South University, Zhuzhou 412000, Hunan, P. R. China.

[2]School of Public Health and Laboratory Medicine, Hunan University of Medicine, Huaihua 418000, Hunan, P. R. China.

[3]Department of Urology, The First Affiliated Hospital, Hengyang Medical School, University of South China, Hengyang, 421200, Hunan, P. R. China.

[#]Contributed equally to this work.

***Correspondence:** Xiao-Liang Xing

xiaoliangxinghnm@126.com

Supplementary: 4 figures and 1 table

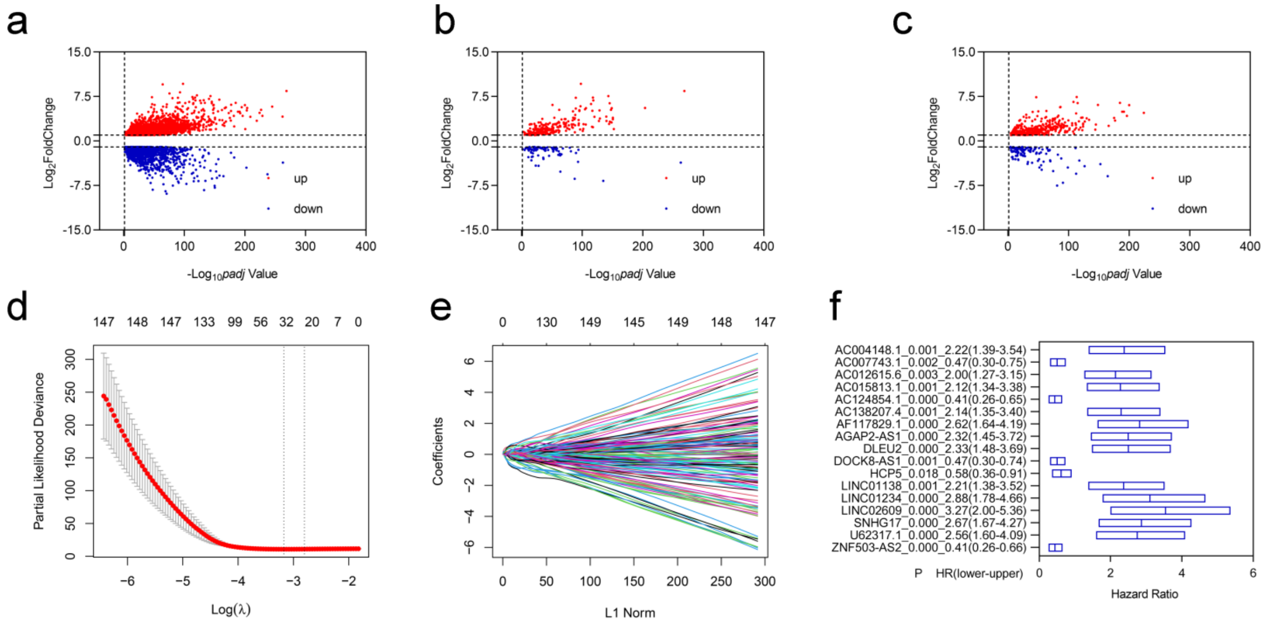

Supplementary Figure 1 Differential expression for KIRC.

a, Volcano of DEGs of KIRC. b, Volcano of FI-DEGs of KIRC. c, Volcano of DELs of KIRC. d, LASSO analyses. f, K–M analysis illustrated 17 FI-DELs were correlated with OS of patients with KIRC followed LASSO analysis.

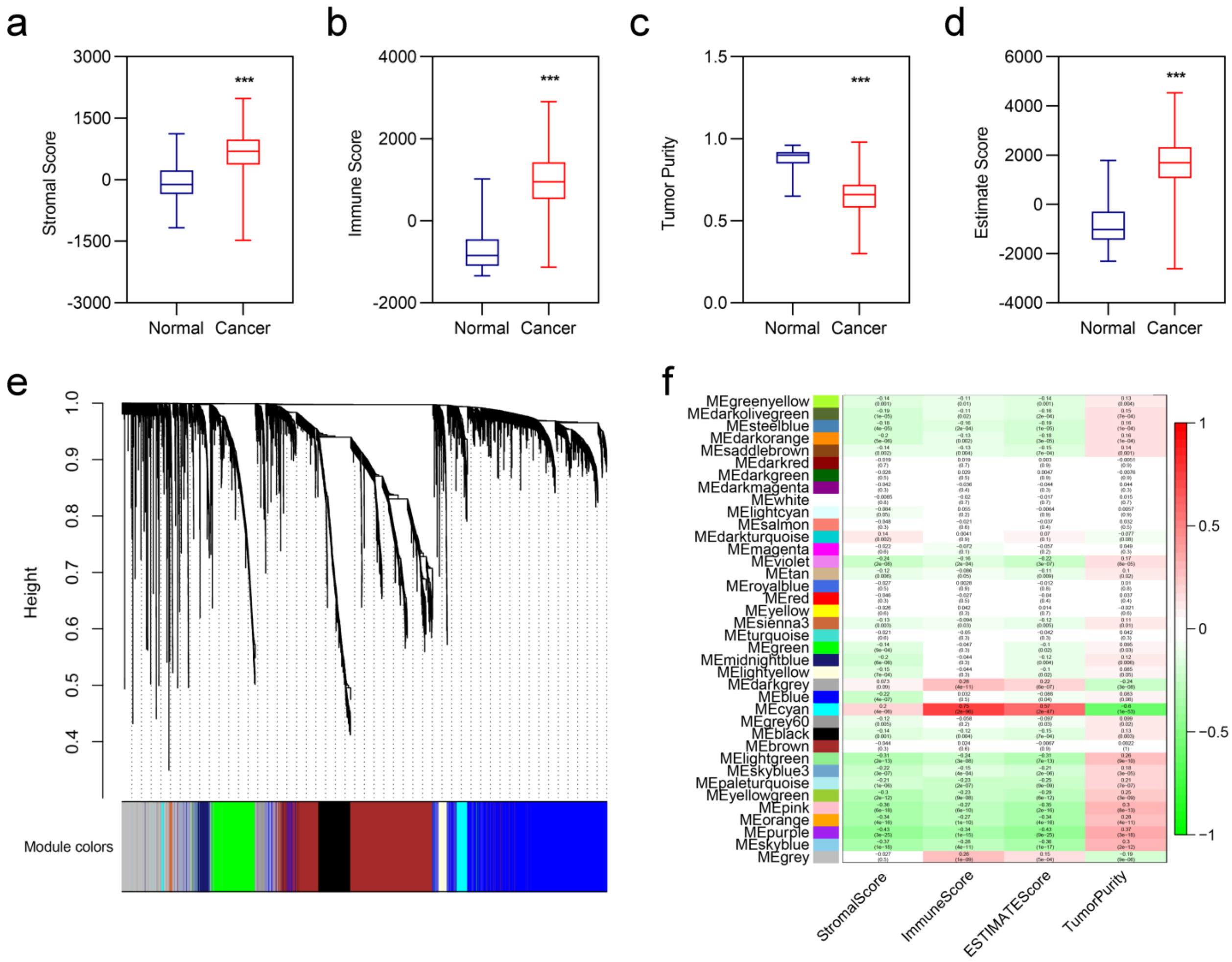

Supplementary Figure 2 The coexpression network established using WGCNA.

a-d, Immune score between patients with KIRC with low CIFI value and patients with KIRC with high CIFI value. a, stromal score. b, immune score. c, tumour purity. d, ESTIMATE score.e-f, 38 modules were obtained by WGCNA.

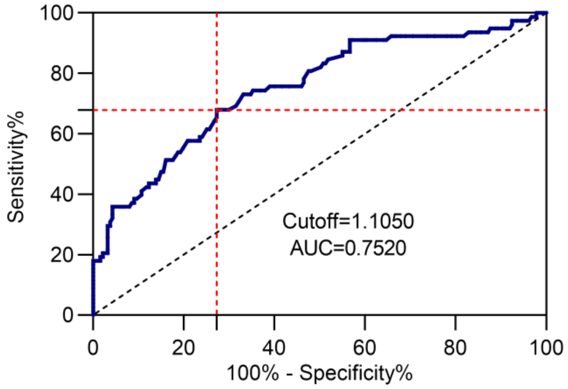

Supplementary Figure 3 The Youden index as optimal cut-off value of CIFI model in the training group.

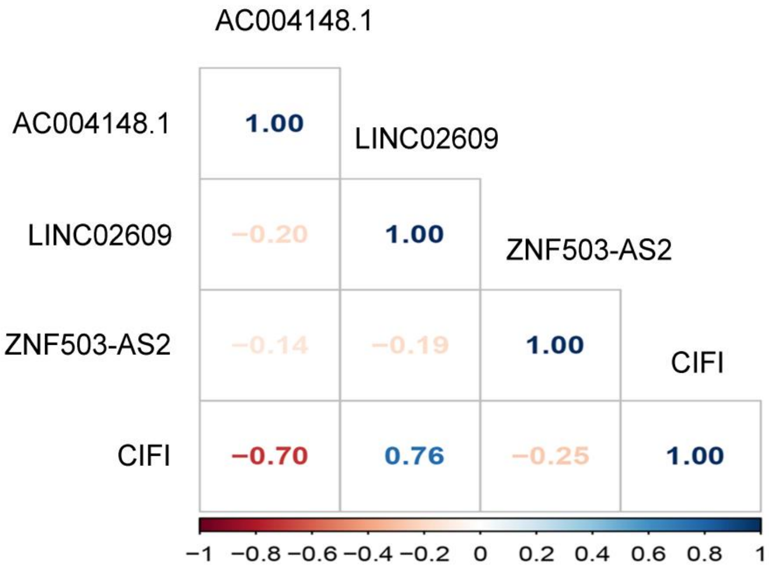

Supplementary Figure 4 Correlation analyses for the candidate prognostic biomarkers with the CIFI value.

Supplementary table 1 Significantly differentially expressed immune cells and immune factors between the normal and patients with KIRC.

Type	Cell	Normal (n=72)		Cancer (n=530)		P
		Mean	SD	Mean	SD	
CIBERSORT	B cell naive	0.09	0.05	0.01	0.02	0.0000
	T cell CD4+ memory resting	0.23	0.06	0.13	0.08	0.0000
	Macrophage M1	0.03	0.02	0.06	0.03	0.0000
	T cell CD8+	0.05	0.04	0.17	0.12	0.0000
	T cell follicular helper	0.01	0.01	0.03	0.03	0.0000
	Mast cell activated	0.05	0.05	0.02	0.03	0.0000
	B cell plasma	0.08	0.06	0.05	0.04	0.0000
	Monocyte	0.08	0.05	0.05	0.05	0.0000
	Macrophage M2	0.26	0.10	0.33	0.12	0.0000
	T cell gamma delta	0.00	0.01	0.02	0.03	0.0000
	T cell regulatory (Tregs)	0.01	0.01	0.01	0.02	0.0008
	NK cell resting	0.00	0.01	0.01	0.02	0.0055
	Macrophage M0	0.00	0.01	0.02	0.06	0.0158
	Eosinophil	0.00	0.00	0.00	0.00	0.0234
	NK cell activated	0.06	0.04	0.05	0.03	0.0255
	Mast cell resting	0.02	0.04	0.01	0.03	0.0392
CIBERSORT-ABS	B cell naive	0.02	0.01	0.01	0.01	0.0000
	Macrophage M2	0.07	0.06	0.18	0.10	0.0000
	Macrophage M1	0.01	0.01	0.03	0.03	0.0000
	T cell CD8+	0.01	0.01	0.10	0.09	0.0000
	T cell follicular helper	0.00	0.00	0.02	0.02	0.0000
	NK cell activated	0.01	0.01	0.03	0.02	0.0000
	T cell regulatory (Tregs)	0.00	0.00	0.01	0.01	0.0000
	T cell gamma delta	0.00	0.00	0.01	0.02	0.0000
	B cell plasma	0.02	0.01	0.03	0.02	0.0009
	Macrophage M0	0.00	0.00	0.01	0.02	0.0048
	NK cell resting	0.00	0.00	0.00	0.01	0.0050
EPIC	T cell CD4+	0.09	0.02	0.04	0.02	0.0000
	Endothelial cell	0.07	0.05	0.19	0.12	0.0000
	Macrophage	0.00	0.00	0.03	0.03	0.0000
	uncharacterized cell	0.79	0.05	0.68	0.14	0.0000
	NK cell	0.00	0.00	0.00	0.00	0.0002
	Cancer associated fibroblast	0.01	0.01	0.03	0.05	0.0014
MCPCOUNTER	T cell	63.42	34.48	10.51	11.49	0.0000
	Monocyte	14.54	8.67	38.57	17.33	0.0000
	Macrophage/Monocyte	14.54	8.67	38.57	17.33	0.0000
	NK cell	0.18	0.17	0.66	0.37	0.0000
	Neutrophil	18.87	6.46	12.07	5.30	0.0000
	cytotoxicity score	1.12	1.33	9.06	7.24	0.0000
	Endothelial cell	24.34	10.13	51.37	30.49	0.0000

	T cell CD8+	1.36	1.42	12.84	17.05	0.0000
	Myeloid dendritic cell	2.78	2.05	4.24	2.51	0.0000
QUANTISEQ	Macrophage M1	0.01	0.01	0.04	0.03	0.0000
	T cell CD4+ (non-regulatory)	0.01	0.01	0.03	0.02	0.0000
	Neutrophil	0.17	0.08	0.12	0.05	0.0000
	T cell CD8+	0.00	0.00	0.03	0.05	0.0000
	Macrophage M2	0.04	0.01	0.06	0.03	0.0000
	uncharacterized cell	0.73	0.06	0.68	0.08	0.0000
	T cell regulatory (Tregs)	0.01	0.01	0.01	0.01	0.0255
	Myeloid dendritic cell	0.02	0.02	0.01	0.02	0.0455
	NK cell	0.01	0.00	0.01	0.01	0.0492
TIMER	Neutrophil	0.03	0.04	0.14	0.10	0.0000
	Myeloid dendritic cell	0.28	0.12	0.50	0.22	0.0000
	T cell CD8+	0.04	0.05	0.21	0.18	0.0000
	T cell CD4+	0.10	0.05	0.14	0.08	0.0001
	Macrophage	0.03	0.06	0.08	0.11	0.0013
	B cell	0.07	0.05	0.10	0.07	0.0032
XCELL	T cell NK	0.01	0.01	0.11	0.05	0.0000
	T cell CD4+ effector memory	0.00	0.01	0.07	0.04	0.0000
	Myeloid dendritic cell activated	0.07	0.08	0.21	0.09	0.0000
	Hematopoietic stem cell	0.47	0.15	0.24	0.15	0.0000
	microenvironment score	0.10	0.05	0.25	0.11	0.0000
	T cell CD4+ Th1	0.09	0.03	0.17	0.07	0.0000
	Endothelial cell	0.10	0.05	0.23	0.12	0.0000
	Monocyte	0.00	0.01	0.04	0.04	0.0000
	immune score	0.01	0.01	0.12	0.11	0.0000
	Mast cell	0.00	0.00	0.01	0.01	0.0000
	T cell CD8+ naive	0.00	0.00	0.01	0.01	0.0000
	T cell CD8+ central memory	0.00	0.01	0.08	0.09	0.0000
	Macrophage	0.00	0.01	0.03	0.04	0.0000
	T cell CD8+	0.00	0.00	0.07	0.09	0.0000
	T cell CD8+ effector memory	0.00	0.00	0.03	0.05	0.0000
	Macrophage M1	0.00	0.00	0.01	0.02	0.0000
	Macrophage M2	0.02	0.01	0.04	0.03	0.0000
	Myeloid dendritic cell	0.00	0.01	0.01	0.01	0.0000
	stroma score	0.09	0.05	0.13	0.07	0.0000
	B cell plasma	0.00	0.00	0.01	0.02	0.0000
	Class-switched memory B cell	0.00	0.00	0.01	0.01	0.0000
	B cell	0.00	0.00	0.02	0.05	0.0000
	Plasmacytoid dendritic cell	0.00	0.00	0.01	0.01	0.0000
	T cell CD4+ central memory	0.00	0.00	0.01	0.01	0.0000
	T cell CD4+ Th2	0.00	0.02	0.03	0.07	0.0004
	Cancer associated fibroblast	0.04	0.07	0.02	0.05	0.0007
	T cell CD4+ naive	0.00	0.00	0.01	0.02	0.0030
	B cell naive	0.00	0.00	0.00	0.00	0.0040

	T cell CD4+ memory	0.00	0.00	0.00	0.01	0.0202
	Common lymphoid progenitor	0.03	0.01	0.03	0.02	0.0269
	Eosinophil	0.00	0.00	0.00	0.00	0.0321
